# Genetic modulation of atrial fibrillation risk in a Hispanic/Latino cohort

**DOI:** 10.1371/journal.pone.0194480

**Published:** 2018-04-06

**Authors:** Brandon Chalazan, Denise Mol, Arvind Sridhar, Zain Alzahrani, Sara D. Darbar, Aylin Ornelas-Loredo, Abdullah Farooqui, Maria Argos, Martha L. Daviglus, Sreenivas Konda, Jalees Rehman, Dawood Darbar

**Affiliations:** 1 Department of Medicine, University of Illinois at Chicago, Chicago, IL, United States of America; 2 Division of Epidemiology and Biostatics, School of Public Health, University of Illinois at Chicago, Chicago, IL, United States of America; 3 Institute for Minority Health Research, University of Illinois at Chicago, Chicago, IL, United States of America; 4 Department of Pharmacology, University of Illinois at Chicago, Chicago, IL, United States of America; Rush University Medical Center, UNITED STATES

## Abstract

Atrial fibrillation (AF) is the most prevalent cardiac rhythm disorder worldwide but the underlying genetic and molecular mechanisms and the response to therapies is not fully understood. Despite a greater burden of AF risk factors in Hispanics/Latinos the prevalence of AF remains low. Over the last decade, genome-wide association studies have identified numerous AF susceptibility loci in mostly whites of European descent. The goal of this study was to determine if the top 9 single nucleotide polymorphisms (SNPs) associated with AF in patients of European descent also increase susceptibility to AF in Hispanics/Latinos. AF cases were prospectively enrolled in the University of Illinois at Chicago (UIC) AF Registry and control subjects were identified from the UIC Cohort of Patients, Family and Friends. AF cases and controls were genotyped for 9 AF risk SNPs at chromosome 1q21: rs13376333, rs6666258; chr1q24: rs3903239; chr4q25: rs2200733; rs10033464; chr10q22: rs10824026; chr14q23: rs1152591; chr16q22: rs2106261 and rs7193343. The study sample consisted of 713 Hispanic/Latino subjects including 103 AF cases and 610 controls. Among the 8 AF risk SNPs genotyped, only rs10033464 SNP at chromosome (chr) 4q25 (near *PITX2*) was significantly associated with development of AF after multiple risk factor adjustment and multiple testing (adj. odds ratio [OR] 2.27, 95% confidence interval [CI] 1.31–3.94; P = 3.3 x 10^−3^). Furthermore, the association remained significant when the analysis was restricted to Hispanics of Mexican descent (adj. OR 2.32, 95% CI 1.35–3.99; P = 0.002. We confirm for the first time the association between a chromosome 4q25 SNP and increased susceptibility to AF in Hispanics/Latinos. While the underlying molecular mechanisms by which the chr4q25 SNP modulates AF risk remains unclear, this study supports a genetic basis for non-familial AF in patients of Hispanic descent.

## Introduction

Atrial fibrillation (AF), the most common sustained arrhythmia worldwide, independently increases the risk of cardiovascular and all-cause mortality, congestive heart failure, and stroke [1]. It was recently estimated that 33.5 million people around the world suffer from AF with a progressive increase in incidence and prevalence over the last 2 decades [2]. While aging of the population may be partly responsible, novel risk factors including race/ethnicity have recently been identified [3]. Despite a higher burden of AF risk factors such as hypertension in African Americans (AAs) and Hispanics/Latinos (H/Ls), the incidence and prevalence of AF is lower than that in whites of European descent [4–7]. Although the etiology of this ‘AF paradox’ in non-Europeans is poorly understood support for a genetic basis has recently been reported [8–10].

Genetic approaches to AF have not only provided important insights into the underlying molecular mechanisms of the arrhythmia but also identified novel therapeutic pathways [3]. Linkage analysis, candidate gene and next generation sequencing approaches have identified mutations in cardiac ion channels, signaling proteins, and sarcomeric proteins linked with early-onset (familial) AF, conduction disease and atrial myopathy [11]. In contrast, genome-wide association studies (GWAS) have consistently identified a locus on chromosome (chr) 4q25 that is associated with AF [12]. The locus is adjacent to the paired-like homeodomain transcription factor 2 gene (*PITX2*), which is critical for pulmonary vein development [13]. A meta-analysis of AF GWAS identified 14 AF risk loci [14, 15] and recently the AFGen Consortium identified an additional 12 novel AF loci [16, 17]. While GWAS have identified over 25 AF loci in mostly whites of European descent, the role of common genetic variation in the pathophysiology of AF in patients of non-European descent remains poorly understood. One study showed that the proportion of European ancestry in AAs was associated with increased risk for AF [18]. A follow-up study using admixture mapping uncovered an AF risk allele on chr10q22 that partially mediated a higher risk for AF in European Americans (EAs) when compared to AAs, and accounted for a modest reduction in developing AF [9]. An earlier study using GWAS AF SNPs identified in EAs, determined if there was an association with AF risk at the chr1q21, chr4q25 and chr16q22 regions in an AA cohort. This study showed that SNPs at these loci increase susceptibility to AF, but that the strongest associations differed from those in subjects of European descent most likely related to linkage disequilibrium (LD) between the race/ethnicities [19].

There is now compelling evidence that AAs and Asians are less likely to develop AF than European whites [20]. Recently, a study conducted in Han Chinese identified an intronic SNP in the *CAV1* gene that was associated with reduced risk for AF after analyzing 6 SNPs previously associated with AF in EAs [21]. Although these studies have provided important insights into underlying mechanisms of AF in whites and AAs, the genetic basis of AF in H/Ls remains unclear. The goal of this study was to determine whether the 9 SNPs most significantly associated with AF in whites of European descent increased susceptibility to the arrhythmia in H/Ls.

## Methods

### Ethics statement

Written informed consent was obtained from all participants under a protocol approved by the University of Illinois at Chicago (UIC) Institutional Review Board.

### Study population

All participants were self-identified as being H/Ls and were over the age of 18 years old in both the UIC AF Registry (UIC AFR) and the UIC Cohort of Patients, Family and Friends (UIC Cohort). Baseline questionnaires detailing study demographics, risk factors, medical history, and blood for DNA extraction was obtained in both clinical-DNA databases. H/L patients with AF (cases) were prospectively enrolled in the UIC AFR established in 2015. All patients had a documented history of AF either by ECG, Holter recording, event monitor, or implantable loop recorders. Any patient that underwent a cardiothoracic surgical procedure and developed AF within 6 months of the surgery was excluded from the study. If AF persisted past 6 months in these patients, they then became eligible for enrollment into the registry. Controls were selected based on enrollment questionnaires detailing self-reporting co-morbidities from the participants in the UIC Cohort that first started enrollment in 2012. The patients were AF-free at the time of recruitment and had at least one chronic disease diagnosis (excluding cancers). The controls, randomly selected from among the UIC Cohort without a history suggestive for AF and at least two 12-lead ECGs showing the absence of AF, were aged (to the 5 years) and sex-matched in ~1:3 ratio.

### Sample collection and processing

Blood samples from consented participants were drawn into one vial. The buffy coat layer containing the white blood cells for DNA extraction was collected from the serum. DNA was extracted using a commercially available kit (Qiagen Puregene, Valencia, CA) and samples were stored in a -80°C freezer until genotyping. Samples were processed according to the Agena iPLEX Gold Protocol. DNA concentrations were between 5–200 ng/ul and aliquots of 2ul were transferred to a 384 well reaction plates for the target specific multiplex PCR amplification. This was followed by shrimp alkaline phosphatase (SAP) treatment, and then a single base extension which was carried out for each multiplex plate. The reaction products were transferred to SpectroCHIP arrays using the RS1000 Nanodispenser. The spectral analysis for single bases when incorporated during extension steps were completed using Matrix Assisted Laser Desorption Ionization—Time of Flight (MALDI-TOF) mass spectrometer and MassARRAY Analyzer software.

### SNP selection and genotyping

We selected the top 9 SNPs most significantly associated with AF and replicated in whites of European descent [14] including those at chr1q21: rs13376333, rs6666258; 1q24: rs3903239, 4q25: rs2200733; rs10033464; 10q22: rs10824026; 14q23: rs1152591; 16q22: rs2106261; rs7193343. Multiplex assay design for genotyping at the 9 SNPs was carried out by Agena Assay Design Suite v2.0. Genotyping calls were made by TyperAnalyzer software using the Autocluster method. Calls were manually reviewed with quality control (QC) and genotype reports in a tabular formation. All SNP calls were >95% with an average call rate of 99%. Eight (3 AF cases, 5 controls) samples were excluded from the analysis due to low genotyping efficiency (<95%). Tests of Hardy-Weinberg equilibrium (HWE) were calculated separately for cases and controls, and no SNP deviated from HWE (P<0.006). While genotyping was performed in all 9 SNPs listed, the rs2106261 SNP in *ZFHX3* was excluded from analysis because the minor allele frequency (MAF) was <5%. Thereby, association analysis was performed in 8 candidate SNPs.

### Statistical methods

Descriptive statistics are presented as mean ± standard deviation (SD) and counts (proportions). We assessed differences between AF cases and controls using a chi-square test or a Student’s t-test for variables listed in Table 1. Genotyping data, assay statistics, and QC parameters for the selected samples were derived from peak area data. All quality matrices including statistics of assay and sample performance across entire dataset were used to exclude poor performing samples and assays from downstream analyses. Individual genotype outputs in a tabular format were combined into a large matrix format for subsequent genetic analysis using PLINK and Golden Helix genome data analyses. Using logistic regression analysis in PLINK association analysis was performed in both unadjusted and adjusted for age, sex, chronic obstructive pulmonary disease (COPD), hypertension (HTN), diabetes mellitus (DM), rheumatic heart disease (RHD), coronary artery disease (CAD), and heart failure (HF) assuming an additive genetic model for all SNPs that passed QC. Correction for multiple comparisons was made by controlling the False Discovery Rate (FDR) using the Bonferroni correction (0.05/8 = 0.0063) to determine the appropriate significant associations in PLINK (22).

**Table 1:** Baseline clinical characteristics and demographics of patients with AF and controls.

	AF (n = 103)	Controls (n = 610)	P-value
Age (SD), years	59±14	46±14	<0.0001
Female	39 (38%)	61 (59%)	<0.0001
Body mass index (kg/m^2^)	32±7.0	31±0.49	0.26
COPD	13 (13%)	19 (3)	<0.0001
Hypertension	70 (68%)	172 (28%)	<0.0001
Diabetes mellitus	35 (34%)	108 (17%)	0.0002
Rheumatic heart disease	17 (17%)	5 (0.82%)	<0.0001
Coronary artery disease	18 (18%)	25 (4.1%)	<0.0001
Heart Failure	29 (28%)	8 (1.3%)	<0.0001
Stroke	4 (0.4%)	11 (1.8%)	0.16

BMI: body mass index; COPD: chronic obstructive pulmonary disease; SD: standard deviation.

## Results

### Baseline clinical characteristics

The study sample consisted of 713 self-identified H/L individuals including 103 AF cases and 610 controls free of AF from the UIC AF Registry and UIC Cohort respectively. The mean age of the AF cases was 59±14 years versus 46±14 years in the controls (**Table 1**). There was a higher prevalence of COPD, HTN, RHD, CAD, HF, and DM in AF cases versus controls.

### Genotyping for AF associated SNPs

The 8 SNPs included in the association analysis were common with MAF ranging from 13–44% (**Table 2**). We showed that rs6666258 on chr1q21, rs10033464 on chr4q25, and rs10824026 on chr10q22 were significantly associated with AF in bivariate analysis (**Table 2**). However, after adjusting for age, sex, COPD, HTN, DM, RHD, CAD, and HF only rs10033464 on chr4q25 (adj. odds ratio [OR] 2.27, 95% confidence interval [CI] 1.31–3.94; P = 3.3 x 10^−3^) was significantly associated with increased risk for AF (**Table 2**). Furthermore, the association remained significant after applying a Bonferroni correction (P<0.0063). As the average age of the controls was significantly less than controls, we age-matched (±4 years) AF cases and controls and showed that there remained a significant association with rs10033464 on chr4q25 after risk factor adjustment (adj. OR 2.48, 95% CI 1.19–5.90; P = 0.015; **S1 Table**). To assess the influence of admixture in our H/L cohort, we performed a sensitivity analysis restricting AF cases (n = 76) and controls (n = 358) to those individuals of Mexican descent only. This showed that there was a significant association between a chr4q25 SNP (rs10033464) and development of AF after adjusting for age and sex (adj. OR 2.32, 95% CI 1.35–3.99; P = 0.002; **S2 Table**).

**Table 2:** Multivariate logistic regression analysis of 8 candidate AF SNPs in patients of Hispanic descent risk factor adjusted.

rsID	Chr.	Gene	Position	Risk/reference allele	MAF (%)	Adj.OR*	95% CI	*P* value	Adj.OR**	95% CI	*P* value
rs13376333	1q21	*KCNN3*	Intronic	T/C	18	0.55	0.34–0.91	0.02	0.57	0.31–1.0	0.061
rs6666258	1q21	*KCNN3*	Intronic	C/G	20	0.51	0.31–0.82	0.0006	0.49	0.28–0.87	0.013
rs3903239	1q24	*PRRX1*	Intergenic	G/A	35	1.28	0.91–1.80	0.16	1.42	0.94–2.13	0.093
rs10033464	4q25	*PITX2*	Intergenic	T/G	13	2.73	1.72–4.33	1.85 x 10^−5^	2.27	1.31–3.94	3.3 x 10^−3^
rs2200733	4q25	*PITX2*	Intergenic	T/C	25	1.12	0.77–1.64	0.54	1.42	0.91–1.58	0.115
rs10824026	10q22	*SYNPO2L*	Intronic	A/G	37	0.65	0.45–0.93	0.01	0.60	0.39–0.91	0.017
rs1152591	14q23	*SYNE2*	Intergenic	A/G	44	0.94	0.68–1.32	0.75	0.87	0.59–1.29	0.49
rs7193343	16q22	*ZFHX3*	Intronic	T/C	43	1.41	1.01–1.96	0.04	1.43	0.95–2.14	0.081

Chr., chromosome; MAF, minor allele frequency; OR, odds ratio; CI, confidence interval.

*Adjusted for age (years) and sex.

** Adjusted for age (years), sex, COPD, hypertension, diabetes mellitus, rheumatic heart disease, coronary artery disease, heart failure, and stroke.

## Discussion

Over the last 15 years, tremendous progress has been made in understanding the genetic basis of AF in whites of European descent. However, the role of common genetic variation in susceptibility to AF in H/Ls remains poorly understood. Here, we show for the first time that a common SNP at the chr4q25 locus (rs10033464) is strongly associated with AF after multiple risk factor adjustment and correction for multiple testing.

There is a fundamental knowledge gap in our understanding of the pathophysiology of AF. Much of our current understanding of the genetic determinants of AF has come from studies performed in mostly whites of European descent. It is now firmly established that despite a greater burden of AF risk factors, non-Europeans are less prone to develop AF when compared to European whites [4–7, 20, 22]. However, whether this AF paradox also applies to patients of Hispanic descent remains unclear. Here, we show for the first time that a common SNP at the chr4q25 locus was strongly associated with AF in H/L patients. Furthermore, this association remained when we restricted analysis to Hispanics of Mexican descent. However, genetic heterogeneity of AF risk loci across race/ethnic groups has recently been reported [23].

Patients of Hispanic descent were at ~2.3-fold increased risk for developing AF after risk factor adjustment if they carried the rs10033464 SNP at the chr4q25 locus. Although the precise mechanism(s) by which SNPs at the chr4q25 modulate AF risk remains unclear [12] the closest gene to the locus is *PITX2*, with the ‘c’ isoform implicated in pulmonary vein development. There is emerging evidence that chr4q25 AF SNPs likely code for a regulatory element that modulates transcription of *PITX2c* [24–26] and signaling pathways that regulate the electrophysiologic substrate for AF with shortening the atrial action potential duration in AF-prone heterozygous mice [27]. Furthermore, there is evidence that atrial fibrosis may also play a critical role in the pathogenesis of AF. *Pitx2c*^-/-^ homozygous mice not only develop 4-chamber cardiac enlargement but also have histological evidence of atrial fibrosis with up-regulation of collagen precursor genes [13]. In contrast, *Pitx2c*^*+/-*^ heterozygous mice show no evidence of cardiac enlargement, but there is increased expression of genes regulating Wnt signaling, a key fibrosis pathway [3, 27, 28]. As the pulmonary veins play a critical role in triggering AF [29], functional studies of PITX2c suggest that modulation of pulmonary vein development may be one common pathway by which chr4q25 SNPs increase susceptibility to AF [13, 30]. Disruption to this pathway results in ectopic electrical activity and propensity to develop AF [30].

Despite AF being the most common arrhythmia requiring drug therapy worldwide responses to therapies especially antiarrhythmic drugs (AADs) are highly variable with ~50% of patients treated with membrane-active drugs experiencing a recurrence of AF within 6 months [31]. Furthermore, AADs carry significant, albeit small, risk for proarrhythmia including drug-induced long QT syndrome. Although genetic approaches to AF have provided important insights into the genetic determinants of AF and identified novel therapeutic targets, the translation of these discoveries to the bedside care of AF patients has thus far been limited. This may be due to the heterogeneity of the underlying electrical substrate, inter-individual differences in disease mechanisms, and our inability to predict individual responses to therapy [3]. Nonetheless, recent advances in our understanding of genetic mechanisms of AF support the hypothesis that variability in response to antiarrhythmic therapy is modulated in part by common AF SNPs. We and others have shown that response to AADs and ablation therapy is modulated by chr4q25 SNPs in whites of European descent [3]. While it remains to be determined if response to AF therapies is modulated by the chr4q25 locus across race/ethnic groups, the findings of this study provide the basis for testing this hypothesis in patients of non-European descent.

On bivariate analysis, 2 SNPs in *KCNN3* (rs13376333 and rs6666258), which encodes the small conductance calcium-activated potassium (SK) channel important for atrial repolarization were associated with reduced risk for AF [32]. Studies performed before the association between *KCNN3* and AF was discovered, showed that burst pacing of pulmonary vein sleeves caused increased trafficking of SK2 to the cell membrane and the associated shortening of the pulmonary vein APs was due to increased SK current (I_SK_) [33]. Additional support for this association comes from SK knockout mice in which prolonged atrial APD and early-afterdepolarizations were record action potential duration (APD) from atrial myocytes [34]. However, it was not until 2010 that Ellinor et al. [35] reported that a common SNP in *KCNN3* was associated with early-onset AF. Over the last few years, a number of studies have shown that inhibition of I_SK_ is antiarrhythmic in isolated heart models of AF [32, 36]. Although the underlying mechanism by which *KCNN3* genetic variants modulate susceptibility to AF is not completely understood, we are the first to show that a common SNP at this locus is associated with reduced risk for AF in H/L patients. We also identified a second SNP on chr10q22 intronic to *SYNPO2L* (synaptopodin 2 like). The protein coded by *SYNPO2L* is expressed in cardiac muscle, localizes to the Z-disc and modulates actin-based shape. Recently, mutations in sarcomeric genes have been linked with early-onset AF and it is possible that a common SNP in myopathy [11]. These “protective SNPs” reduced the relative AF risk by approximately 50%, which is quite substantial. Identification of such SNPs that reduce the risk of AF could not only lead to a better understanding of the AF paradox in non-Europeans but also identify novel therapeutic targets to prevent AF disease progression. However, it should be appreciated that after adjustment for multiple AF risk factors, SNPs in *KCCN3* and *SYNPO2L* no longer remained significant.

This study has a number of limitations. First, the number of AF cases in our sample was relatively small. This is likely a reflection of the observed reduced prevalence of AF among H/Ls as compared with European-descent individuals. We had 72% power to replicate the 8 SNPs previously associated with AF in EAs with 103 AF cases and 610 controls assuming a MAF of 10% and an OR ≥2.0. It is possible that the lack of an association between the 7 other SNPs associated with AF in European whites may relate to the limited number of AF cases. Second, the age of the controls was significantly younger than the AF cases. As the UIC Cohort mostly consists of normal volunteers with minimal or no comorbidities, matching all AF cases with controls was challenging. However, we did age-match (±4 years) 103 AF cases and 103 controls and showed that there remained a significant association with rs10033464 SNP on chr4q25 after multiple risk factor adjustment (**S1 Table**). Third, it is possible that common AF SNPs may vary across the diverse H/L sample included in this study; such heterogeneity across race/ethnicities has recently been reported [23]. Another explanation for why a European AF SNP did not replicate in a H/L sample is because the LD structure varies across race/ethnicities. It is possible that the identified SNP in whites may not be as strongly correlated with the true underlying causal SNP in H/Ls. Third, we did not estimate admixture in cases and controls in our primary analysis. However, as this is a relatively new cohort, GWAS data or ancestry informative markers are not currently available. Importantly though >75% of cases and controls in our cohort are individuals of Mexican descent, which is consistent with the Hispanic population in Chicago. Our sensitivity analysis showed that a significant association between a chr4q25 SNP and AF remained when the analysis was restricted to AF cases and controls of Mexican descent (**S2 Table**). Fourth, AF ascertainment was not identical in AF cases and controls. However, participants in the UIC Cohort undergo an extensive cardiovascular assessment including detailed history and physical examination. The controls used in this study gave no history suggestive for AF and a minimum of 2 electrocardiograms (ECGs) failed to document AF. Fourth, the OR of 2.3 is higher than previously reported in other race/ethnicities cohorts raising the possibility of cryptic relatedness, confounding or genotyping error. We have reviewed the AF cases and controls and found no evidence of relatedness across the cohorts. Fifth, we selected SNPs focused on genetic targets thought to be directly associated with AF and SNPs associated with AF risk factors were not evaluated.

Overall, the P-values presented here and in the S1 Table were not corrected for multiple testing and FDR [37, 38]. The Bonferroni correction was not applied to the majority of the analyses in this study as it is considered overly conservative for datasets with low levels of LD between SNPs. However, the rs10033464 SNP on chr4q25 survived Bonferroni correction (adj. OR 2.27, 95% CI 1.31–3.94; P = 3.3 x 10^−3^) in our H/L sample. Replication of the associations presented here in an independent dataset is warranted given the limited sample sizes and variability observed when correcting for multiple testing. However, because the prevalence of AF is relatively low in H/Ls identifying a replication cohort is challenging especially one of Mexican descent. It is important however to emphasize that the UIC AF Registry is one of the largest cohort of AF patients of Mexican descent.

In conclusion, we confirm for the first time the association between a chr4q25 SNP and increased risk for the development of AF in patients of Hispanic descent. This finding needs to be replicated in larger Hispanic cohorts of Mexican descent. While future studies also need to identify the underlying molecular mechanisms by which chr4q25 SNPs modulate AF risk, this is one of the first reports of a genetic basis for non-familial AF in patients of Hispanic descent.

## Supporting information

S1 Table Multivariate analysis of 8 candidate AF SNPs in 103 age-matched (±4 years) AF cases and 103 age-matched controls (without AF) of Hispanic descent with adjustment for AF risk factors. (DOCX)

S2 Table Multivariate regression analysis of 8 candidate AF SNPs in 76 AF cases and 358 controls (without AF) in Hispanics of Mexican descent with adjustment for multiple AF risk factors. (DOCX)
