# Supplementary material for: Genetic modulation of atrial fibrillation risk in a Hispanic/Latino cohort
Source: PLoS One. 2018 Apr 6;13(4):e0194480. doi: 10.1371/journal.pone.0194480 (PMC5889061; doi:10.1371/journal.pone.0194480)
Supplement: S1 Table — (DOCX) [file pone.0194480.s001.docx]

**S1 Table.** Multivariate analysis of 8 candidate AF SNPs in 103 age-matched (±4 years) AF cases and 103 age-matched

controls (without AF) of Hispanic descent with adjustment for AF risk factors.

| rsID | Chr. | Gene | Position | Risk/reference  allele | MAF  (%) | Adj. OR* | 95% CI | *P* value | Adj. OR** | 95% CI | *P* value |
| --- | --- | --- | --- | --- | --- | --- | --- | --- | --- | --- | --- |
| rs13376333 | 1q21 | *KCNN3* | Intronic | T/C | 18 | 0.55 | 0.31-0.95 | 0.035 | 0.67 | 0.34-1.35 | 0.267 |
| rs6666258 | 1q21 | *KCNN3* | Intronic | C/G | 20 | 0.29 | 0.30-0.93 | 0.026 | 0.63 | 0.31-1.28 | 0.203 |
| rs3903239 | 1q24 | *PRRX1* | Intergenic | G/A | 35 | 1.28 | 0.82-1.98 | 0.275 | 1.83 | 1.05-3.18 | 0.032 |
| rs10033464 | 4q25 | *PITX2* | Intergenic | T/G | 13 | 2.77 | 1.48-5.18 | 1.4 x 10^-3^ | 2.48 | 1.19-5.90 | 0.015 |
| rs2200733 | 4q25 | *PITX2* | Intergenic | T/C | 25 | 1.16 | 0.74-1.81 | 0.51 | 1.19 | 0.70-2.03 | 0.514 |
| rs10824026 | 10q22 | *SYNPO2L* | Intronic | A/G | 37 | 0.94 | 0.61-0.14 | 0.78 | 0.90 | 0.53-1.52 | 0.702 |
| rs1152591 | 14q23 | *SYNE2* | Intergenic | A/G | 44 | 1.03 | 0.69-1.54 | 0.85 | 0.93 | 0.57-1.59 | 0.775 |
| rs7193343 | 16q22 | *ZFHX3* | Intronic | T/C | 43 | 1.42 | 0.91-2.22 | 0.12 | 1.62 | 0.95-2.78 | 0.079 |

Chr., chromosome; H/L, Hispanic/Latino; MAF, minor allele frequency; OR, odds ratio; CI, confidence interval.

*Adjusted for sex; **Adjusted for sex, chronic obstructive pulmonary disease (COPD), hypertension (HTN), diabetes mellitus

(DM), rheumatic heart disease (RHD), coronary artery disease (CAD), heart failure (HF), and stroke.
