# Supplementary material for: Genetic modulation of atrial fibrillation risk in a Hispanic/Latino cohort
Source: PLoS One. 2018 Apr 6;13(4):e0194480. doi: 10.1371/journal.pone.0194480 (PMC5889061; doi:10.1371/journal.pone.0194480)
Supplement: S2 Table — (DOCX) [file pone.0194480.s002.docx]

**S2 Table.** Multivariate regression analysis of 8 candidate AF SNPs in 76 AF cases and 358 controls (without AF) in

Hispanics of Mexican descent with adjustment for multiple AF risk factors.

| rsID | Chr. | Gene | Position | Risk/reference  allele | MAF  (%) | Adj. OR* | 95% CI | *P* value | Adj. OR** | 95% CI | *P* value |
| --- | --- | --- | --- | --- | --- | --- | --- | --- | --- | --- | --- |
| rs13376333 | 1q21 | *KCNN3* | Intronic | T/C | 14 | 0.71 | 0.37-1.35 | 0.298 | 0.78 | 0.34-1.76 | 0.557 |
| rs6666258 | 1q21 | *KCNN3* | Intronic | C/G | 14 | 0.71 | 0.37-1.35 | 0.302 | 0.78 | 0.34-1.76 | 0.990 |
| rs3903239 | 1q24 | *PRRX1* | Intergenic | G/A | 37 | 0.91 | 0.60-1.39 | 0.662 | 1.00 | 0.58-1.73 | 0.560 |
| rs10033464 | 4q25 | *PITX2* | Intergenic | T/G | 15 | 2.32 | 1.35-3.99 | 0.008 | 1.87 | 0.92-3.79 | 0.024 |
| rs2200733 | 4q25 | *PITX2* | Intergenic | T/C | 26 | 0.90 | 0.57-1.23 | 0.661 | 1.15 | 0.65-2.02 | 0.631 |
| rs10824026 | 10q22 | *SYNPO2L* | Intronic | A/G | 34 | 0.78 | 0.49-1.23 | 0.285 | 0.79 | 0.43-1.44 | 0.449 |
| rs1152591 | 14q23 | *SYNE2* | Intergenic | A/G | 49 | 1.48 | 0.96-2.26 | 0.072 | 2.14 | 1.25-3.91 | 0.006 |
| rs7193343 | 16q22 | *ZFHX3* | Intronic | T/C | 38 | 1.48 | 0.98-2.24 | 0.060 | 1.41 | 0.80-2.49 | 0.223 |

*Adjusted for sex; **Adjusted for age (years), sex, COPD, HTN, DM, RHD, CAD, HF, and stroke.
